# Comprehensive Planning of Laboratory Equipment Based on Genetic Algorithms

**DOI:** 10.1155/2022/5242251

**Published:** 2022-09-12

**Authors:** Tiantian Mi

**Affiliations:** School of Innovation and Entrepreneurship, Shandong Women's University, Jinan, Shandong 250300, China

## Abstract

Laboratory equipment planning is a very important task in modern enterprise management. Laboratory equipment planning by computer algorithm is a very complex NP-hard combinatorial optimization problem, so it is impossible to find an accurate algorithm in polynomial time. In this study, an improved genetic algorithm is used to solve and analyze the comprehensive planning of laboratory equipment. After analyzing the traditional heuristic algorithm and genetic algorithm to solve the simple laboratory equipment planning problem, the simple laboratory equipment planning is designed and implemented according to the principle of the heuristic algorithm. Finally, the algorithm is implemented in Python. A general equipment planning based on genetic algorithm with two selection operators is realized. Two constraints of test start and completion time are given. In the scenario of using multiple test equipment for a test project, the possible solutions of laboratory equipment planning under given constraints are analyzed. The efficiency coefficient is not necessarily a constant, it is related to the output characteristics of energy equipment. Three independent planning algorithms are used to solve the actual test requirements. One is the planning method based on manual test scheduling in the test cycle of experimental instruments, the other is the genetic algorithm for gene location crossover operator, and the third is the genetic algorithm for experimental part crossover operator. The planning solutions obtained by the three algorithms are compared from three aspects: the shortest time to complete the test, the calculation time of the algorithm, and the utilization of the test equipment.

## 1. Introduction

Modern large enterprises as well as large laboratories in society have numerous experimental equipment, and there are usually a variety of tests that the experimental equipment can perform, and the specific use of the experimental equipment is arranged to match the needs of the customer [1]. The customer's test application requirements usually include the type of test, the length of the test, and the start time of the test. The majority of laboratory equipment is currently managed by manual scheduling, which no longer meets the needs of companies as the number of laboratory test equipment increases, the number of test types increases and customer needs increase. Internet is running in a steady state, and the load rate of energy equipment is in a high range. The problem of laboratory equipment planning itself has not been extensively studied in laboratory equipment planning, which refers to the scheduling of many part experiments and test equipment to meet specific constraints, to enable the overall planning to achieve the overall objective of reaching an optimum. The optimal value of this measure obtained is used as the planning solution; it first transforms the problem into an integer planning model defining an objective function and solves for the set of variables that make the objective function optimal under the given constraints [2]. The genetic algorithm inherits the advantages of the genetic algorithm itself, i.e., the idea and process of solving the optimization problem while using the quantum characteristic to compensate for the lack of population diversity, making the algorithms spatial searchability and convergence speed improved, achieving better optimization results than the traditional genetic algorithm. Genetic algorithms are widely used in different types of optimization problems, and their computational performance is better than traditional algorithms, solving a wide range of practical problems in different disciplines. The core operation of quantum genetic algorithms is the evolutionary update of quantum rotating gates, but traditional quantum genetic algorithms use a fixed rotation angle strategy and the rotation direction of quantum gates is determined according to a look-up table, which involves the judgment of several conditions and affects the convergence speed of the algorithm.

The basic idea of its search is to use the evaluation function to evaluate the current search state and find the most promising node to expand. If the evaluation function is used at each step of the search to rank and select the nodes for possible expansion, the algorithm is known as the A-algorithm, which has high computational complexity [3]. Therefore, it is easy to fall into the local optimal solution when solving complex function problems. The genetic algorithm is inspired by Darwin's theory of biological evolution, and the entire algorithm operates in a similar way to human evolution, by adaptively searching for the optimal value of the problem through parental gene selection, mutation, reproduction, and evolutionary mechanisms. Although the basic principles of genetic algorithms are very simple, they are very effective in providing an efficient method of optimization search, avoiding complex mathematical models and operations, so they are quickly gaining a great deal of research and application [4]. Genetic algorithms have the characteristics of autonomous selection, autonomous optimization, autonomous adaptation, and population evolution, and are particularly suitable for large-scale complex optimization and non-linear optimization problems that are difficult to solve with traditional search algorithms.

Newly evolved populations inherit the best genes from the previous generation of populations and repeat the above operations to reproduce and optimize, with the fitness of the chromosomes in the population increasing and getting closer and closer to the approximate optimal solution of the problem until certain decision conditions are met and the operation is terminated [5]. Genetic algorithms are simple in structure and operation and can be used to obtain a globally optimal solution to the problem. To enrich population diversity and evolutionary process diversification, solve the defect that some local spaces cannot be searched. The actual solution to a laboratory equipment planning problem is a very complex problem, so it is necessary to simplify the actual problem and give a solution, and then extend and implement the solution under real constraints and circumstances. In the smart manufacturing digital workshop, there is a close relationship between multirow equipment layout and AGV path planning. The equipment layout process considers the amount of AGV transport and the reserved AGV travel path, while the equipment layout results will directly affect the AGV path planning. Nowadays, most of the research studies these two problems in isolation, and after the equipment layout is completed, the path planning of the AGV is then considered, without considering the inner connection between the two, making the result, not the optimal solution that can be achieved, resulting in lower production efficiency. The rationality of the equipment layout and the efficiency of the AGV path planning together affect the productivity of the entire digital workshop for intelligent manufacturing, and there are huge economic benefits. Considering both equipment layout and AGV path planning in the layout of the workshop can more comprehensively improve the flexibility of the manufacturing system and reduce costs. The problem of multi-row equipment layout with integrated AGV path planning is a problem of research value.

## 2. Related Works

A hybrid algorithm based on variable neighborhood search and ant colony optimization is proposed to solve the single-row equipment layout problem, which utilizes three-domain structures to improve the operational efficiency of the algorithm and reduce the mathematical computation of the objective function. Wang et al. propose a permutation-based genetic algorithm to solve the single-row equipment layout problem. The genetic operator is improved to optimality by regularizing and randomizing the genetic operator and using an improved crossover operator and variational operator [6]. Kari et al. assume that the length and width of the devices are not predetermined and model the length and width of each device as a bounded variable for this uncertainty, introducing new parameters to achieve a robust approach [7]. A new adaptive algorithm is designed to determine the robust layout according to the needs of the decision-maker [8]. The development of big data technology has led to a deeper analysis of energy information data, which can help and guide the construction, operation, and maintenance of integrated energy systems through the analysis of data and information flows. With the release of State Grid's New Energy Cloud, Green State Grid Platform, and Guodian Group's Tianshu No.1, the development of integrated energy systems has entered the data era [9]. Integrated energy systems can realize information interaction, and resource sharing and explore optimization potential with the help of big data and Internet technologies [10]. The content that meets the requirements is reused, and the content that does not match and does not meet the requirements is adjusted and corrected. These platforms can coordinate the construction and operation of integrated energy system projects in various regions and guide the new construction of integrated energy system projects.

A rational mathematical description of the quantum genetic algorithm is provided, and to solve the problem that the algorithm tends to fall into local optimum solutions, the algorithm introduces quantum catastrophe. Through simulation experiments, it confirmed that the convergence accuracy of the quantum genetic algorithm based on the disaster factor is better than that of the traditional quantum genetic algorithm [11]. A cloud quantum genetic algorithm is proposed for application to crane bridge design. To improve the convergence accuracy of the algorithm, the algorithm optimizes the initialization coding, changes the crossover and variation methods using the X-conditional cloud generator, and adjusts the quantum gates, and the convergence accuracy of the algorithm is significantly improved [12]. Based on the analysis of the shortcomings of the traditional quantum genetic algorithm, an adaptive quantum genetic algorithm based on small habitats is proposed, which is improved by using the sine and a cosine encoding method, small habitat initialization population, determining the quantum gate rotation direction and adaptive rotation angle strategy, as well as quantum convergence gate and quantum catastrophe [13]. The adaptive quantum genetic algorithm for small habitats is verified through numerical experiments to further improve the convergence speed while maintaining the convergence accuracy of the algorithm.

The optimal planning method obtains planning solutions by constructing and solving planning optimization models, but the method requires accurate modeling, which increases the complexity of the models, and the difficulty of solving them. The usual methods are automatic system correction and human interaction correction. Currently, planning models for multienergy systems usually have economic optimization as the optimization objective. With the energy cost minimization of the thermal, gas, and electronic subsystems as the multi-level optimization objective, the energy balance of the distributed integrated energy system, and the operational characteristics of the energy equipment as constraints, a distributed energy system capacity optimization allocation model is constructed and solved using a region shrinkage algorithm. The simple laboratory equipment planning problem is a special case of the laboratory equipment planning problem. Compared with the simple laboratory equipment planning problem, the general laboratory equipment planning problem is relatively more complex and has higher flexibility, as well as a wider range of applications.

## 3. Genetic Algorithm Design Analysis

The genetic algorithm is inspired by Darwin's theory of biological evolution, and the entire algorithm is like human evolution in that it adaptively searches for the optimal value of the problem through parental gene selection, mutation, reproduction, and evolutionary mechanisms. Although the basic principles of genetic algorithms are very simple, they are very effective in providing an efficient method of optimization search, avoiding complex mathematical models and operations, so they are quickly gaining a great deal of research and application [14]. As a result, many researches are very different from theoretical research in the actual production process. Genetic algorithms have the characteristics of autonomous selection, autonomous optimization, autonomous adaptation, and population evolution, and are particularly suitable for large-scale complex optimization and non-linear optimization problems that are difficult to solve with traditional search algorithms. For these reasons, genetic algorithms have been developed in the field of computer technology and have been successfully used in many areas such as image processing, optimal control, machine learning, and artificial intelligence.

The merit of a chromosome is expressed by the value of the corresponding chromosome fitness, which is evaluated by a series of algorithmic operations of selective duplication, crossover, and mutation to filter the chromosomes so that those with high fitness remain [15]. Chromosomes with low fitness eliminated from the population and a new population of chromosomes are generated. The newly evolved population inherits the best genes from the previous generation and repeats the above operations to reproduce and optimize the population. The fitness of the chromosomes in the population increases and gets closer and closer to the approximate optimal solution of the problem until a certain decision condition is met and the operation is terminated. Genetic algorithms are simple in structure and operation and can be used to obtain a globally optimal solution to the problem.

The objective of the simple laboratory equipment planning problem is to obtain an equipment planning solution that minimizes the time to complete all experiments, which can be expressed in terms of *P*_max_. Suppose that the planning solution of the simple laboratory equipment planning problem, i.e., the sequence of experiments for the part is {*L*_1_, *L*_2_, ......, *L*_*L*_}, and the time for the part *L*_*i*_ to complete the corresponding test item on the test equipment *s* can be denoted as *P*(*L*_1_, *s*). The completion time *P*_max_ of the planned program can then be derived from the following analysis. The design scheme does not meet the production requirements of the workshop, resulting in economic losses for the company.

(1) $$\begin{array}{c} P\left(L_{1},s\right)=T_{L_{1}}s\text{,} \end{array}$$

*L*_1_ is the first part to be tested in the equipment planning scheme, and this equation represents the time for the first test item of part *L*_1_ to be completed on the relevant test equipment.

(2) $$\begin{array}{c} P\left(L_{1},s\right)=P\left(T_{L_{1}},s-1\right)-T_{L_{1}}s\text{.} \end{array}$$

The time for the *s*-th test item of part *L*_1_ to complete testing on the associated test equipment, which is equal to the actual time for the *s*-1st test item of the experimental part to complete testing on the associated test equipment, plus the time it takes for the *s*-th test item to complete testing on the associated test equipment.

(3) $$\begin{array}{c} P\left(L_{1},s\right)=P\left(T_{L_{1}},1\right)+T_{L_{1}}s\text{.} \end{array}$$

**Figure 1:**
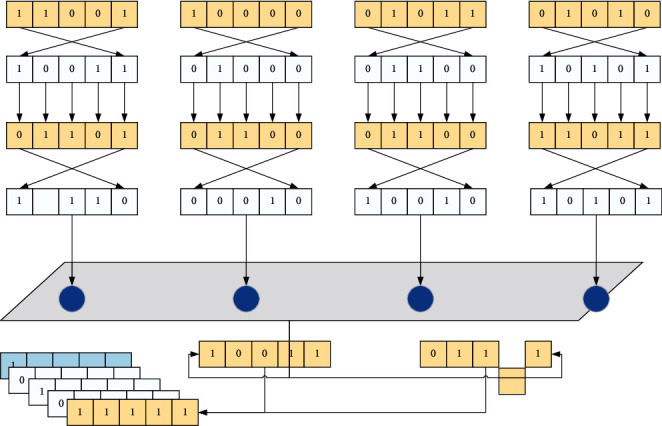
Genetic algorithm framework.

The time to complete the test on the relevant test equipment for the *s*-th test item of the experimental part *L*_1_ is equal to the greater the time to complete the test on the relevant test equipment for the s-th test item of the experimental part *L*_1_-1 and the time to complete the test on the relevant test equipment for the s-th test item of the experimental part *L*_1_ and the test time required for the s-th test item of the experimental part *L*_1_, as shown in Figure 1. That is to say, each part test is constrained by the test order, and the test order of different test items cannot be changed at will; and when a certain time of a certain test equipment is allocated to a specific test item.

The quantum genetic algorithm uses quantum revolving gates, which are unique to quantum theory, to update populations and enrich population diversity by changing the probability magnitude of quantum states through the interaction of individual quantum superposition states. It cannot be scheduled for this test equipment within this time period. Quantum rotational gates are indispensable in quantum genetic algorithms because they ensure the convergence of quantum states to individuals with higher fitness, according to the quantum rotational gate look-up table [16]. The transformations performed by the quantum gates are all transformations in Hilbert space, ensuring that the quantum superposition states still satisfy the normalization condition after the quantum gate action.

(4) $$\begin{array}{c} R\left(\theta \right)=\left[\begin{array}{cc} \cos\;\theta & \sin\;\theta \\ -\sin\;\theta & \cos\;\theta \end{array}\right]\text{.} \end{array}$$

The process by which different forms of energy flow through the energy conversion chain can be divided into two steps: energy distribution and energy conduction or conversion. Energy distribution describes the proportional distribution of various forms of energy input to different energy conduction devices or energy conversion devices; energy conversion describes the process of converting one or more forms of energy to other forms of energy-by-energy conversion devices; energy conduction describes the process of transporting the same form of energy through energy conduction devices without passing through energy conversion devices.

(5) $$\begin{array}{c} P^{out}+ES=\lambda \eta \left(P^{in}-ES\right)\text{.} \end{array}$$

The distribution factor is a controllable variable and is usually used as a decision variable in the optimization of the operation of the campus energy network. The efficiency coefficient, on the other hand, is not necessarily constant and is related to the output characteristics of the energy equipment. When the campus energy Internet is in steady-state operation and the load factor of the energy equipment is in a high range, the efficiency coefficient can be approximated to remain constant, thus realizing the linearization of the mathematical model of the above energy power balance relationship. Must be completed by a specific time. The solution to this problem is to add a constraint to the requirements of ordinary laboratory experiment planning.

The robust optimization model described in equation (5) requires that the constraints are satisfied in any scenario within the uncertain budget set and that the resulting solution is highly conservative. To address the problem of the strong conservativeness of the traditional robust optimization model, a weak robust optimization model is proposed, in which slack variables are added to the constraints to allow for constraint violations, but the degree of constraint violations cannot exceed a finite value, and the model can effectively improve the conservativeness of traditional robust optimization.

(6) $$\begin{array}{c} a_{i}^{T}x+\gamma_{i}^{2}\geq \overset{J}{\underset{j=1}{\sum }}k_{k}\left(b_{j}-\xi_{j}b_{j}\right)\text{.} \end{array}$$

It is trapped in a local optimal solution and adopts a fixed rotation angle strategy, the rotation direction needs to be determined by checking the table, and the process involves several judgment conditions, which affect the efficiency of the algorithm. To address these problems, an improved approach to the traditional quantum genetic algorithm is proposed, which is based on the adaptive rotation angle strategy of the small habitat strategy, constructing a determinant A to determine the rotation direction, which does not involve multiple judgment conditions of table look-up, and improves the convergence speed of the algorithm. Facing the drawback of premature convergence of quantum bits, which leads the algorithm to fall into local optimal solutions, and premature maturity, two strategies, *H*_*ξ*_ convergence gate, and quantum catastrophe, work together to increase the search space of the algorithm, prevent it from falling into local optimal solutions, and improve the convergence accuracy of the algorithm [17].

**Figure 2:**
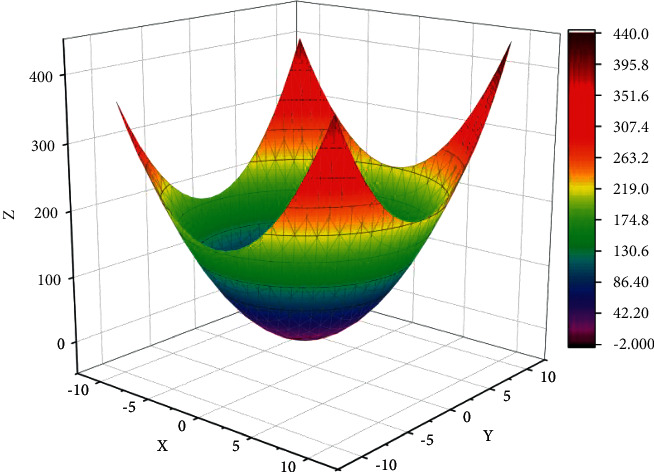
Rosen rocks function grid.

As many individuals gather near the same point in the late stage of evolution, it may lead to some local spaces not being searched and new individuals cannot be evolved, and thus it is easy to fall into local optimal solutions when solving complex function problems. To enrich population diversity and diversify the evolutionary process, the defect that some local spaces cannot be searched for is solved. With the increase in laboratory test equipment, the increase in test types, and the increase in customer demand, this management method can no longer meet the needs of enterprises. A small habitat strategy is used to distribute individuals evenly throughout the solution space, i.e., the population is partitioned into several subpopulations, each subpopulation is iteratively searched for optimality, and finally, the best individuals from different subpopulations are formed into a new population for subsequent search, as shown in Figure 2.

When using the example inference technique, the key features of the problem to be solved are first described and the key elements of the problem are translated into a form that can be recognized by the example library. The instance retrieval process is one of the most important aspects of the instance reasoning technique. The process is designed to give solutions to new problems, reuse content that meets the requirements, and adjust and revise content that does not match and does not meet the requirements, usually using methods such as automatic system revision and human interaction. Case preservation means that the revision process evaluates the proposed solution and if the retrieved case successfully solves the problem, the new solution is retained and stored as a new case. The magnitudes of the objective functions are generally different from each other and the importance of the objective functions cannot be compared, so how to set the weights is a more difficult problem to solve. The second method is to solve multiobjective optimization problems through meta-heuristic algorithms, such as genetic algorithms, particle swarm algorithms, firefly algorithms, fish swarm algorithms. At the same time, quantum characteristics are used to make up for the deficiency of insufficient population diversity, which improves the spatial search ability and convergence speed of the algorithm, and achieves a better optimization effect than the traditional genetic algorithm.

For general multiobjective optimization problems, when the solution range is wide, the solution can be solved in two ways. The first is to convert the multiobjective problem into a single-objective problem. There are three ways to achieve this conversion: the linear weighted combination method, the ideal point method, and the principal objective function method [18]. *T* A set of Pareto solutions can be obtained by intelligent algorithms. In this Pareto solution set, each solution cannot dominate and be dominated over the others, and each solution can be considered a valid solution relative to this optimization problem.

In digital workshops, equipment layout and AGV path planning are the two most important elements that affect the cost and productivity of the entire workshop in practice, and AGV path planning and workshop equipment layout are not independent problems, they are strongly coupled. However, many researchers have overlooked the connection that exists between these two issues and has studied them separately, which has resulted in many studies being very different from the theoretical studies in the actual production process, and the design solutions do not meet the production requirements of the workshop, resulting in economic losses for the company. This section analyses the relationship that exists between equipment layout and AGV path planning, and integrates considerations to determine the specific layout of the workshop, providing theoretical support for equipment layout and AGV path planning for the workshop.

## 4. Laboratory Equipment Integrated Planning Model Analysis

When the laboratory is arranging tests, the test arrangement is completely dependent on the time to get the tested part, which in practice is dynamically changing. At the same time, the laboratory is also faced with equipment damage or repair, urgent insertion of test tasks and test failures, etc. Experimental test planning is highly dynamic and stochastic. Constraint means that test scheduling must follow a defined sequence of test items and a guideline for prioritizing resource usage. This means that each part test is bound by the test order and the sequential test order of different test items cannot be switched at will; and when a certain time slot of test equipment is allocated to a specific test item, other tests cannot be scheduled to this test equipment within this time slot.

In laboratory management, experimental requirements come from different departments and customers, which may have different importance to laboratory management, and often the experiments yield conflicting test objectives. In practice customers require the shortest test completion times, departments require the least number of overruns, and management will require the highest utilization of test equipment. Find the most promising node to scale. If the evaluation function is used at each step of the search to sort the nodes that may expand. Laboratory management should therefore plan laboratory testing to meet these objectives as closely as possible to achieve the best possible benefits for the company and the laboratory [19].

**Figure 3:**
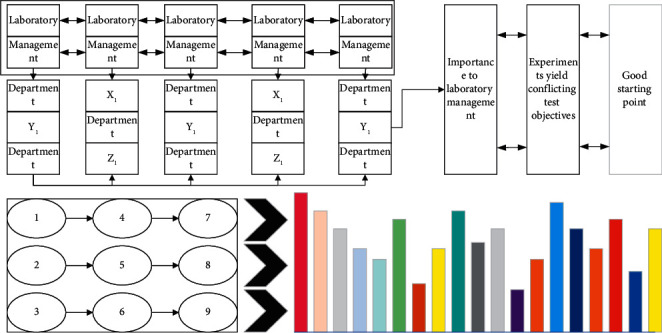
Schematic of improved segmentation in an equipment group layout.

In practical laboratory test planning, it is a common scenario to specify the completion time of a part: for example, when the results of a certain test are the basis for a product development timeline and the continuation of a product development project depends on the results of that test; or when the results of a certain test are the basis for a major company decision and must be completed by a specific time. The solution to this problem is to add a constraint to the requirements of ordinary laboratory experiment planning. Although the basic principle of genetic algorithm is very simple, it is very effective to provide an efficient method for optimizing search, and avoiding complex mathematical models and operations. Another more common constraint in practical laboratory test planning is to specify a start time for a part to be tested. Usually, this is because the part may be ready by a specific time point and it is required that the part be tested immediately to schedule it according to the optimal planning, as shown in Figure 3.

Typically for such stochastic uncertainty problems, converting the uncertainty into a deterministic problem can be solved by building a probabilistic model. The main difficulty in establishing a probability model is determining the probability density function, which is commonly found in normal, logarithmic, and uniform distributions. Uncertain variables are transformed into random variables that obey a certain probability distribution to be solved. In probabilistic methods, the probability density function of a variable is difficult to find out precisely and usually requires a lot of experiments and is derived from many statistics. In practical engineering, however, we do not need to establish the probability density function of a variable exactly. To describe the distribution of a random variable, we can use the expectation and variance, two important statistical features, to describe it.

A gene chain encoded by a staging cache-based layout has a staging cache at the back of the device group represented by the gene at the location corresponding to the gene's position on the device-group-based layout gene chain, and a staging cache does not exist for a gene with an ordinal number of 0. The fourth gene in the gene chain encoded by the staging cache layout, with an ordinal number of 1, corresponds to the fifth gene in the gene chain encoded by the device-based group layout, i.e., represents the presence of a staging cache after device group 9.

**Figure 4:**
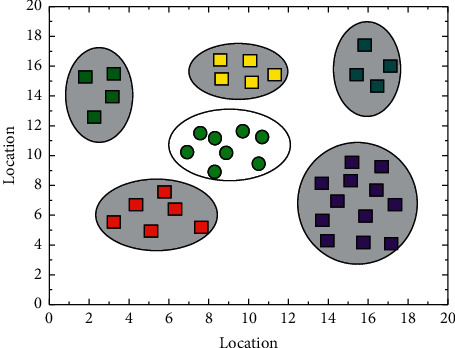
Optimal solution distribution.

A staging buffer cannot be placed after the first device group, the penultimate device group, and the penultimate device group. If a staging cache zone exists, the number of device groups in the zone will be less than two, which does not comply with the zoning rules. The length of the gene chain encoded in the staging buffer layout can therefore be reduced to *n*-3. This unequal double-stranded chromosome not only makes subsequent gene chain operations faster, reducing both the time and space complexity of the algorithm but also allows for the rapid elimination of non-compliant individuals, as shown in Figure 4. It is then extended and implemented to solve under real constraints and environments.

The selection process moves the algorithm in the direction of the optimal solution and allows for a wide distribution of populations. The selection operator increases the probability of high-performance individuals being selected, which in turn increases the convergence and computational efficiency of the algorithm. After the nondominated ranking and crowding calculations, everyone is given two attribute values, nondominated ranking, and crowding, and the binary tournament method is used to select the individuals of the parent generation that require crossover variation.

The nondominance ranking of the contemporary population is calculated and the portion of individuals with a low nondominance hierarchy, which has high fitness values, are selected to find the common approximate subsequence of the genes of these individuals. If available, this fraction of genes is extracted as a vaccine, placed in the vaccine pool, and the vaccine pool is updated [20]. In the digital workshop of intelligent manufacturing, there is a close relationship between multi-line equipment layout and AGV path planning. Vaccination selects individuals from the current population with a high nondominance hierarchy, selects some of these individuals, and vaccinates that individual with a randomly selected vaccine from the vaccine bank, adjusting the gene sequencing to make the gene sequence valid, like the previous mutation operation of unequal double-stranded chromosomes, to obtain a new individual.

## 5. Analysis of Results

### 5.1. Algorithm Performance Analysis

**Figure 5:**
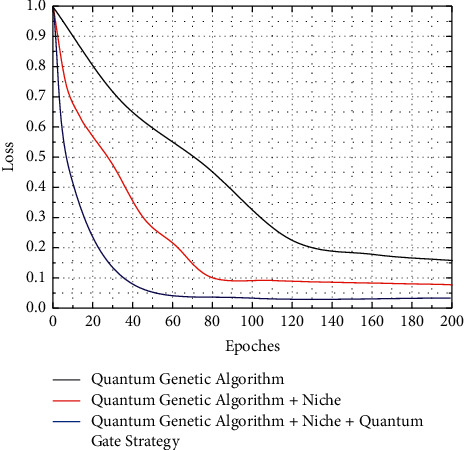
Comparison of the Rosen rock's function algorithm.

Figure 5 shows that the quantum genetic algorithm incorporating the coevolution strategy for small habitats converges faster than the traditional quantum genetic algorithm. The average fitness of the Rosen rock's function was 0.00649 and 0.00335, and the average fitness of the Schaffer function was 0.9943 and 0.9974, respectively. The average fitness of the Rosen rock's function was 0.00649 and 0.00335, and the average fitness of the Schaffer function was 0.9943 and 0.9974, respectively. The average number of iterations for the Rosen rock's function was 78.5 and 56.4, and the average number of iterations for the Schaffer function was 61.5 and 37.6, further demonstrating the short evolutionary time and fast convergence of the quantum genetic algorithm incorporating the small habitat strategy. The effectiveness and efficiency of incorporating the habitat let strategy, i.e., fast convergence and high convergence accuracy, were verified by the above two test functions. The fitness of chromosomes in the population increases continuously and gets closer and closer to the approximate optimal solution of the problem, until certain conditions are met to terminate the operation.

The convergence speed of the quantum genetic algorithm with the addition of the quantum gate strategy on top of the small habitat strategy is further improved. The average fitness of the Rosen rocks and Schaffer functions are 0.00219 and 0.9985 respectively, and the average evolutionary generation of the two functions are 39.2 and 21.4 respectively. The quantum genetic algorithm with a quantum gate strategy on top of the small habitat strategy has obvious advantages in terms of convergence accuracy and convergence speed, demonstrating the effectiveness and efficiency of its application in function optimization.

Quantum convergence gates are used to prevent the quantum bit evolution process from collapsing prematurely and causing the chromosome to fail to evolve, and quantum catastrophes are used to find the global optimal solution by allowing the algorithm to jump out of the local solution space and search the global space after the algorithm has already fallen into a locally optimal solution. To see their impact on the function, we use the Rosen rocks and Schaffer functions to analyze separately traditional quantum genetic algorithms and quantum genetic algorithms incorporating convergence gates and quantum catastrophe strategies.

The quantum genetic algorithm with the quantum convergence gate and the quantum catastrophe strategy has a higher convergence accuracy. The average fitness of the Rosen rocks and Schaffer functions are 0.00143 and 0.9993, respectively, and the average evolutionary algebra is 92.5 and 69.4. Compared with the derived traditional quantum genetic algorithm, the quantum genetic algorithm with the quantum convergence gate and quantum catastrophe strategy has a higher convergence accuracy. The quantum genetic algorithm with the quantum convergence gate and quantum catastrophe strategy resulted in a closer approximation to the optimal solution than the conventional quantum genetic algorithm, but the evolutionary algebra did not improve. The reason for this is that the use of quantum convergence gates increases the time complexity of the algorithm, making convergence slower and that the occurrence of quantum catastrophes, which improve the optimal solution, does not reduce the evolutionary generation, but even increases it.

**Figure 6:**
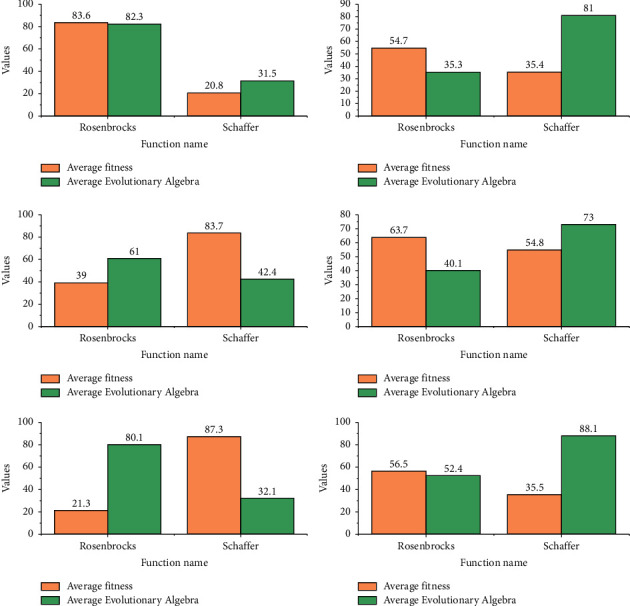
Statistical results of the average fitness and evolutionary algebra of the improved algorithm.

As shown in Figure 6, from the graph of the evolutionary convergence process of the test functions and the statistics of the fitness and evolutionary generations, the convergence speed and accuracy of the small habitat-based adaptive quantum genetic algorithm incorporating quantum convergence gates and quantum catastrophes are significantly better than those of the traditional quantum genetic algorithm. The convergence speed of the Rosen rock's function and the Schaffer function is improved by 41.656% and the convergence speed of the Rosen rocks and Schaffer functions improved by 41.656% and 42.602%, respectively, which is lower than that of the algorithm without the quantum convergence gate and quantum catastrophe, but the inclusion of the quantum convergence gate and quantum catastrophe also made the average fitness closer to the optimal value.

The simulation experiments show that the improvements such as the initialization of the small habitat population, the determination of the rotation direction of the quantum gate, and the angle adjustment of the quantum gate speed up the convergence of the function and require fewer average evolutionary generations, reflecting a strong solving capability and a small improvement inaccuracy. The introduction of the *H*_*ξ*_ gate and the quantum catastrophe improves the optimal solution and the algorithm is less likely to fall into a local optimum, but it is clear from the average evolutionary generations that the *H*_*ξ*_ gate and the quantum. The introduction of the *H*_*ξ*_ gate and the quantum catastrophe significantly reduces the convergence speed of the algorithm. It is finally verified that the addition of quantum convergence gates and quantum catastrophes to the small habitat-based adaptive quantum genetic algorithm not only improves the convergence speed of the algorithm but also makes the convergence accuracy of the algorithm closer to that of the optimal solution.

### 5.2. Results of the Integrated Planning Model

**Figure 7:**
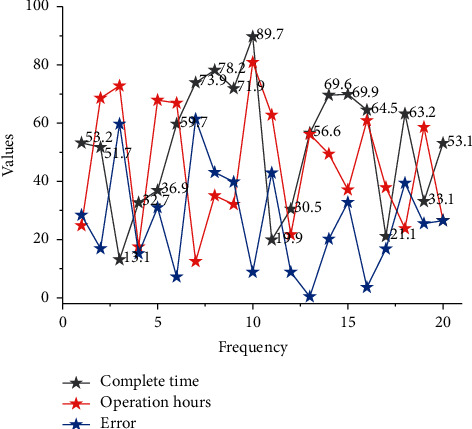
Comparison of the two algorithms given a test start time.

Based on the above analysis, the fitness function of the general laboratory experimental planning was recorded and two corresponding coding schemes were obtained. It assumed that the input conditions for the laboratory experimental planning remain the same, while the parameters of the genetic algorithm remain the same. Based on all known inputs and constraints, the two fitness functions are encoded into the genetic algorithm and the two sets of results are obtained after five separate operations, as shown in Figure 7.

The two encodings above only modify the fitness function, while the body of the genetic operation and the inputs to the operation remain unchanged, so there is no difference in the convergence of the results or the optimal solution obtained. They both solve equipment planning problems by specifying the test start time for certain parts. However, comparing the two codes, it is faster to operate with the test equipment occupancy method. Therefore, the test equipment occupancy method is used in the design of practical genetic algorithms. In practical laboratory management, there are often multiple test devices for the same test item. Laboratory equipment planning requires a balanced and efficient use of all test equipment, to complete all parts in the shortest possible time while making full use of the equipment.

The uniqueness of the coding scheme of the original common laboratory equipment planning algorithm is because the test item of a part and its corresponding test equipment are unique. Energy conversion describes the process in which energy conversion equipment converts one or more forms of energy into one or more other forms of energy; energy transfer describes the process of the same form of energy not passing through energy conversion equipment, but through energy transmission equipment delivery process. So, it is possible to express both the test equipment and the test item in terms of the number of times the part recurs. To achieve the same uniqueness, a relatively straightforward solution is to add the test equipment identification code to the original code. In contrast to ordinary laboratory equipment planning, the same test item has multiple test equipment planning given an application scenario where *m* test equipment is tested for the same test item, so much so that a chromosome code cannot correspond to a unique planning solution. To achieve this without changing the chromosome code, a uniform criterion for test device allocation can be developed after the genetic algorithm has been computed. This test equipment allocation criterion is used to decode the resulting optimal solution.

**Figure 8:**
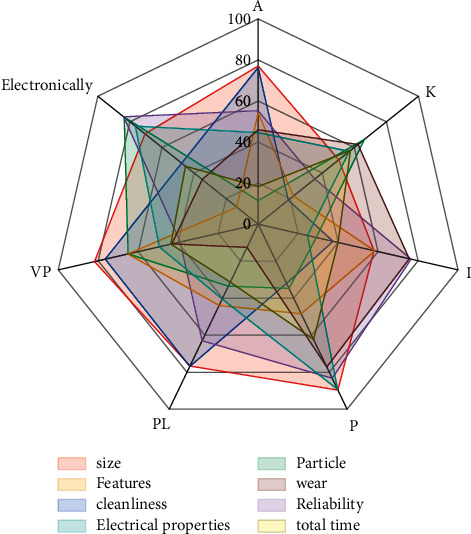
Test times for each test item for a part.

Relying on the empirical planning of the laboratory supervisor, it is common to prioritize the testing of certain parts requiring a specified time to start the experiment, and then consider testing parts requiring a longer total test time. For parts with a relatively short total test time, the experiments required for these parts are planned sequentially after the first two types of parts have been scheduled. This is based on a simple planning approach that lacks scientific rigor and does not make the most efficient use of all the laboratory equipment, as shown in Figure 8.

As products differ, the test items required for their testing vary considerably, and some experimental items are not required for specific parts. In this case, the test time for the test items that not required can be set to 0. The time taken to complete all tests is very long and the equipment is less utilized and more time-consuming. Therefore, manual scheduling based on priority levels does not give a more efficient planning solution and a more efficient algorithm must be used.

Manual planning of experiments based on the specific requirements of a given test requires a lot of time, careful manual scheduling, and a unique experimental planning solution. However, because it is manual, the chance of error is still very high. By using a genetic algorithm, a more satisfactory plan can be obtained by simply running the genetic algorithm after all the parameters and code have been completed. The two genetic algorithms in this chapter can also be run more times or the parameters of the genetic algorithm can be improved to obtain a more optimized planning solution.

The main reason for the low utilization of the test equipment in the manual scheduling scheme is that when scheduling manually, the test equipment requiring a given start time is first arranged and then sequentially arranged according to the test cycle. So, there are multiple tests with more spread-out test items, resulting in lower equipment utilization. In contrast, the two genetic algorithms result in a solution where the test items of the equipment are completed in a more concentrated time, so the test equipment utilization is naturally higher. When comparing the test equipment utilization rates of the two genetic algorithms, the experimental part crossover operator genetic algorithm solution has a higher average test equipment utilization rate of nearly 3% than that obtained by the gene location crossover operator genetic algorithm. However, considering the total test completion time of the solutions, the genetic algorithm for gene location crossover arithmetic is still the preferred recommended solution for laboratory equipment planning.

## 6. Conclusion

In this study, the encoding and decoding methods of general equipment planning, and the corresponding encoding and decoding methods are proposed. The initial population generation method is defined, the fitness function algorithm is written, and various crossover operators are analyzed. Finally, two selection operators are designed. The study of this is a work of great theoretical importance and strong application. First, the traditional heuristic algorithm and genetic algorithm for solving the simple equipment planning problem are analyzed, and simple laboratory equipment planning is designed and implemented based on the principles of the heuristic algorithm. For solving laboratory equipment planning with given constraints, two possible solutions were analyzed: solving by generating a compliant initial population and solving by weighting the fitness function to eliminate noncompliant genes. The genetic algorithm with a weighting of the fitness function is the feasible and preferred solution, and the associated fitness function is designed. The planning solutions obtained by the three algorithms were compared in terms of the minimum time to complete the test, the computation time of the algorithm, and the utilization of the test equipment. The genetic algorithm with gene location crossover operator is the most optimized solution for laboratory equipment planning.

## Data Availability

The data used to support the findings of this study are available from the corresponding author upon request.
